# *Serratula coronata* L. Mediated Synthesis of ZnO Nanoparticles and Their Application for the Removal of Alizarin Yellow R by Photocatalytic Degradation and Adsorption

**DOI:** 10.3390/nano12193293

**Published:** 2022-09-22

**Authors:** Anastassiya A. Mashentseva, Nurgulim A. Aimanova, Nursanat Parmanbek, Bakhtiyar S. Temirgaziyev, Murat Barsbay, Maxim V. Zdorovets

**Affiliations:** 1 The Institute of Nuclear Physics of the Republic of Kazakhstan, Almaty 050032, Kazakhstan; 2 Department of Chemistry, L.N. Gumilyov Eurasian National University, Nur-Sultan 010008, Kazakhstan; 3 Department of Chemistry, Buketov Karaganda University, Karaganda 100024, Kazakhstan; 4 Department of Chemistry, Hacettepe University, Ankara 06800, Turkiye

**Keywords:** biogenic nanoparticles, wet combustion synthesis, zinc oxide, alizarin yellow R, fotocatalysis, adsorption, dye removal, *Serratula coronata* L.

## Abstract

In this study, the potential of biogenic zinc oxide nanoparticles (ZnO NPs) in the removal of alizarin yellow R (AY) from aqueous solutions by photocatalytic degradation, as well as adsorption, was investigated. The synthesized ZnO NPs were prepared by the simple wet-combustion method using the plant extract of *Serratula coronata* L. as a reducing and stabilizing agent and characterized by powder X-ray diffraction, scanning electron microscopy, energy dispersive X-ray and X-ray photoelectron spectroscopy. Photocatalytic degradation of AY was monitored by UV–visible spectroscopy and the effects of parameters, such as light source type (UV-, visible- and sunlight), incubation time, pH, catalyst dosage and temperature on degradation were investigated. It was demonstrated that the source of light plays an important role in the efficiency of the reaction and the UV-assisted degradation of AY was the most effective, compared to the others. The degradation reaction of AY was found to follow the Langmuir-Hinshelwood mechanism and a pseudo-first-order kinetic model. The degradation kinetics of AY accelerated with increasing temperature, and the lowest activation energy (*E_a_*) was calculated as 3.4 kJ/mol for the UV-light irradiation system, while the *E_a_* values were 4.18 and 7.37 kJ/mol for visible light and sunlight, respectively. The dye removal by the adsorption process was also affected by several parameters, such as pH, sorbent amount and contact time. The data obtained in the kinetics study fit the pseudo-second-order equation best model and the rate constant was calculated as 0.001 g/mg·min. The isotherm analysis indicated that the equilibrium data fit well with the Freundlich isotherm model. The maximum adsorption capacity of AY on biogenic ZnO NPs was 5.34 mg/g.

## 1. Introduction

Over the last two decades, zinc oxide nanoparticles (ZnO NPs) have been the object of close attention due to their unique properties and high performance. In modern laboratory practice, a variety of physical, chemical and combined synthetic methods are used to produce ZnO NPs of different morphologies and sizes [1,2,3]. Although many of these methods yield excellent results in terms of physical and morphological properties and size, the production of nanomaterials for medical and pharmacological purposes additionally requires the meeting of bioavailability, safety and efficacy standards. For this reason, efficient and “green” synthesis methods, that ensure the use of synthesized NPs, especially in the mentioned application areas, are of great interest. In this regard, one of the most promising groups of synthesis methods are biological green chemistry methods that use plant and animal objects, as well as various enzymes and microorganisms, as highly effective reducing agents [4,5]. At the heart of most biogenic methods in which NPs are synthesized using plant materials, a metal salt reacts with a plant extract and the reaction is completed in a relatively short time at ambient temperature. The undeniable advantages of green chemistry methods for producing “biogenic” nanoparticles are the simplicity of the methodology, environmental friendliness, high reactivity, purity, and low toxicity of the final product [6,7].

The high biocompatibility of ZnO nanoparticles, along with their cytotoxic, antibacterial, and fungicidal activities, ensure their large-scale use in medicine [8,9]. Along with titanium dioxide, ZnO-based nanomaterials are among the most frequently used in photocatalysis due to the high thermal conductivity, high exciton binding energy (60 meV), high electron mobility and wide bandgap (3.2–3.4 eV) of zinc oxide [10,11]. In a number of studies, ZnO nanoparticles have demonstrated high catalytic and sorption activity in the reactions of removing nitrophenol [12,13], metal ions [14,15,16,17], antibiotics [18] and organic dyes [19,20,21].

All modified alizarin dyes (alizarin red C, alizarin yellow P and alizarin yellow GG) are widely used in the textile industry and also as indicators. Alizarin in wastewater is not biodegradable and causes significant damage when released into the environment. The development of new types of materials that enable the effective removal of this dye, which is known for its high toxicity and strongly pronounced carcinogenic properties, is of interest and demand. Practically, catalytic degradation and sorption methods are most commonly used for the removal of alizarin dye [22], although different methods, such as gamma irradiation, have also been studied [23]. The high efficiency of nanoscale catalysts for the removal of alizarin yellow P from aqueous solutions under UV light [24,25,26], and Nd:YAG laser [27] have been previously demonstrated. Sorbents based on nanoparticles, including biogenic ones, supported by attachment to large substrates of different origins, have been widely used to achieve removal of alizarin dyes [23,24].

The novelty of this research is the obtaining of ZnO NPs using a plant extract of the aerial part of *Serratula coronata* L., grown in Central Kazakhstan, as well as a comprehensive application of these biogenic nanoparticles in the photocatalytic degradation and sorption of alizarin yellow (AY) dye. Since the synthesis method is based on plant extracts, the obtained biogenic ZnO NPs are also promising for medical or pharmaceutical applications. *Serratula coronata* L. is a polycarpic, small-rooted, sympodial herbaceous perennial with semi-rosette above-ground shoots [25]. At least 14 phenolic compounds (7.3%), ten of which can be classified as flavonoid glycosides and aglycones (apigenin, luteolin, quercetin and their glycosides), were found in the above-ground part of *Serratula coronata* L., cultivated in Siberia [26]. In addition, 3-O-methoxyquercitin, 4-*β*-D-glucosides of luteolin and quercetin have also been isolated [27]. Most of the flavonoids in the aerial part of the plant are concentrated in the leaves (14.87–18.5%), much less in inflorescences (4.18–5.88%) and minimally in stems (2.61–3.44%), and vary by location and year of collection [28,29]. We have previously found that the above-ground part of *Serratula coronata* L., grown in Central Kazakhstan, also contains significant amounts of phenolic compounds, including flavonoids [30].

In this research, we studied the degradation of AY under the influence of different light sources and determined not only the optimal reaction conditions, but also the main thermodynamic characteristics of biogenic ZnO NPs as catalyst. The physicochemical conditions of the removal of AY by sorption, such as pH, dye concentration and sorption time were optimized, allowing a detailed elucidation of the kinetic and thermodynamic aspects and mechanism of dye sorption. The results obtained with this study underline the potential of biogenic NPs obtained by using sustainable sources for environmental protection.

## 2. Materials and Methods

### 2.1. Materials and Reagents

Analytical reagent grade zinc nitrate hexahydrate, alizarin yellow, petroleum ether (all Sigma Aldrich), isobutanol (Component-Reactive LLC, Moscow, Russia) and ethanol (Talgar-Spirt LLP, Almaty, Kazakhstan) were used without additional purification. Deionized water (18.2 Mohm/cm, Aquilon-D301, Aquilon, Podolsk, Russia) was used in all experiments.

### 2.2. Preparation of the Plant Extract

The collection of *Serratula coronata* L. was carried out at the vegetation phase in mid-May 2021. The raw material collection area was in Spasskie Sopky of the Abay district of the Karaganda region (Central Kazakhstan region). The dry raw materials (buds, leaves and stems) of *Serratula coronata* L. were crushed to a particle size of 2–3 mm, and the weight of the sample for each extraction experiment was 20 g. To obtain the maximum polyphenolic component extraction, a 70% (*v*/*v*) water-ethanol solution was used, which ensured the maximum amount of flavonoids from plant raw materials [31].

**Figure 1:**
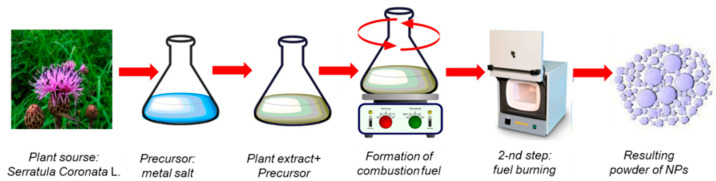
Schematic representation of obtaining biogenic ZnO nanoparticles via wet combustion approach.

Zinc oxide nanoparticles were obtained via wet combustion, as summarized in Figure 1, by the following procedure: 1.0 g of *Serratula coronata* L. extract and 4.02 g of Zn(NO_3_)_2_∙6H_2_O were dissolved in 25 mL of water-ethanol mixture under constant stirring, then the solution was placed in a ceramic crucible in a muffle oven preheated to 200 °C for combustion for 3 min. The resulting mixture was filtered to remove ash from the plant extract and thoroughly washed several times with deionized water to remove any remaining impurities. The resulting mixture was then annealed in a muffle oven at 600 °C for 2 h. The resulting fine white powder, weighing 3.77 g, was stored in a sealed container and used without further purification.

### 2.3. Study of the Structure and Composition of Nanoparticles

Morphological examinations and dimensional measurements of the resulting nanoparticles were performed using a JEOL JFC-7500F scanning electron microscope (SEM) (Tokyo, Japan) and JEOL JEM-1400Plus transmission electron microscope (TEM) (Tokyo, Japan). Energy-dispersive X-ray spectroscopy (EDS) measurements were carried out using a Hitachi TM3030 (Hitachi Ltd., Chiyoda, Tokyo, Japan) microscope with a Bruker XFlash MIN SVE (Bruker, Karlsruhe, Germany) microanalysis system at an accelerating voltage of 15 kV.

The crystal structure of the nanoparticles was examined on a D8 Advance diffractometer (Bruker, Karlsruhe, Germany) in the angular range of 2θ 10–80° with a step of 2θ = 0.02° (measuring time: 1 s, tube mode: 40 kV, 40 mA). The mean size of crystallites was determined via the broadening of X-ray diffraction reflections using the Scherrer formula [32]. The phase composition was determined using the Rietveld method, which is based on approximating the areas of the diffraction peaks and determining the convergence with reference values for each phase [33]. The volume fraction of the phase was determined using Equation (1) [34]:

(1) $$V_{\mathrm{admixture}}=\frac{RI_{\mathrm{phase}}}{I_{\mathrm{admixture}}+RI_{phase}},$$

where *I*_phase_ is the average integral intensity of the main phase of the diffraction line, *I*_admixture_ is the average integral intensity of the additional phase, and *R* is the structural coefficient equal to 1.

XPS measurements were carried out using a Thermo Scientific K-Alpha spectrometer (Waltham, MA, USA) with a monochromatized Al Kα X-ray source (1486.6 eV photons) at a constant dwell time of 100 ms, pass energy of 30 eV with a step of 0.1 eV for core-level spectra and 200 eV with a step of 1.0 eV for survey spectra. The pressure in the analysis chamber was maintained at 2 × 10^−9^ Torr or lower. The binding energy (BE) values were referred to the C 1s peak at 285 eV. Processing of the data was carried out using Avantage software (version 5.41, 2019, Waltham, MA, USA)

The charge on the adsorbent surface, depending on pH, was investigated by determining the pH_zpc_ value in the pH range from 3.0 to 9.0, according to the method described in [35]: 10 mL of NaCl solution (0.01 M) was brought to the desired pH value (pH_i_) by adding 0.1 M of HCl or NaOH. Then, 50 mg of ZnO nanopowder was added to each flask and shaken on a shaker (IKA KS 3000i, Konigswinter, Germany) at room temperature for 12 h. The NPs were removed from the solution by filtration and the final pH (pH_f_) of the filtrate was measured using a pH-meter (HI2020-02, HANNA Instruments, Smithfield, VA, USA).

### 2.4. Photocatalytic Degradation of AY

A certain amount (between 10 to 100 mg) of zinc oxide catalyst powder was suspended in 100 mL of pre-determined concentration of dye solution in ethanol–water mixture (50% *v*/*v*) and then stirred intensively for 60 min in the dark to achieve adsorption equilibrium in the “catalyst-dye” system. A 300 W high-pressure UV-lamp (Ultra-Vitalux 300 W, Osram, Augsburg, Germany) was used as the source of UV-light. In the case of visible light, a 500 W linear halogen lamp with a UV cut-off filter (Econur, Vito) was used. The distance from the light source to the working solution was 15 cm. Experiments under sunlight were conducted on a sunny day in May, between 1 p.m. and 3 p.m., without using any additional light sources. In all cases, a 1.0 mL reaction mixture was taken every 30 min and the optical density was determined on a Specord-250 spectrophotometer (Jena Analytic, Jena, Germany) in the wavelength range 200–800 nm. The degree of dye degradation (D%) was determined according to Formula (2):

(2) $$D=\frac{C_{0}-C_{t}}{C_{0}}\times 100\%=\frac{A_{0}-A_{t}}{A_{0}}\times 100\%$$

where C_0_ and C_t_ are AY concentrations, and A_0_ and A_t_ are absorbances at 373 nm at the beginning and time t, respectively.

The effect of temperature on the dye degradation efficiency was studied under similar conditions in the temperature range of 10–45 °C.

In order to examine the effect of catalyst mass on dye degradation efficiency, the nanoparticle mass ranged from 10 to 100 mg, the concentration of alizarin yellow was 1.0 mg/L, and the exposure time of the mixture in all experiments was 60 min.

The influence of the initial feed concentration of the dye on its degradation efficiency was investigated in the concentration range of 3.0–30.0 mg/L; the amount of catalyst loaded in all experiments was 50 mg.

When examining the reproducibility of the results obtained, at the end of each test cycle the catalyst was precipitated by centrifugation at 15,000 g for 15 min, washed with deionized water, dried at 50 °C and used in the next cycle.

### 2.5. Study of Removal of AY by Sorption

All experiments conducted to determine the AY adsorption performance of biogenic ZnO NPs were conducted in batch mode. Adsorption kinetics were studied at an AY concentration of 1.0 mg/mL (pH 7.0). Disposable plastic vials (Isolab, Eschau, Germany) containing 15.0 mL of AY solution and 50 mg of NPs were shaken at 100 rpm (IKA KS 3000 IS, Konigswinter, Germany) for different times between 15 min and 6 h at room temperature. Each experiment was repeated in triplicate. The concentration of AY in aliquots was determined using the calibration curve (y = 0.058x + 0.0343 *R*^2^ = 0.997). The amount of AY adsorbed was calculated using Equation (3) [36]:

(3) $$\;Q_{e}=\frac{\left(C_{0}-C_{e}\right)\times V}{m}$$

where *Q_e_* is the amount of AY adsorbed by the unit mass of ZnO (mg/g), C_0_ is the feed concentration (mg/L), C_e_ is the concentration of AY in aliquots (mg/L), *V* is the volume of the solution (L), and *m* is the amount of ZnO NPs used (g).

The percent dye removal (% 𝑅) was calculated from Equation (4):

(4) $$R=\frac{\left(C_{0}-C_{e}\right)}{C_{0}}\times 100\%$$

where C_0_ is the feed concentration (mg/L), C_e_ is the concentration of AY in aliquots (mg/L).

The effect of pH on AY adsorption was studied in the pH range of 3 to 9, keeping other parameters constant (initial AY concentration: 20 mg/L; adsorbent dose: 50 mg; contact time: 120 min). The pH of the solution was adjusted dropwise with 1.0 N HCl_(aq)_ and 1.0 N NaOH_(aq)_. and measured using a digital pH meter (HANNA HI2020-02, Smithfield, VA, USA). All experiments were performed in triplicate.

## 3. Results

### 3.1. Synthesis and Structural Characterization of ZnO NPs

**Figure 2:**
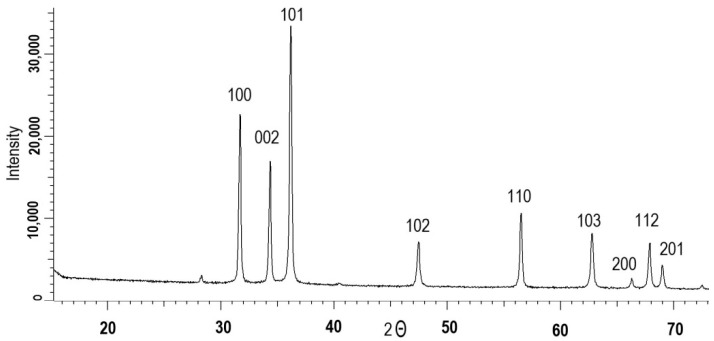
X-ray diffraction (XRD) pattern of the synthesized biogenic ZnO NPs.

In order to obtain information on the crystallographic and chemical structure, along with physical characteristics, of biogenic ZnO NPs, a variety of analyses were carried out. The X-ray diffractogram of the synthesized nanoparticles in Figure 2 identified ZnO phase-specific diffraction peaks at 2θ = 32.54° (110), 35.58° (002), 38.86° (200), 40.43° (112), 48.57° (−202), 53.65° (020), 58.39° (202), 61.57° (−113) and 68.07° (113) [37,38,39]. The defined planes were in accordance with the JCPDS file (JCPDS: 01-007-2551) corresponding to the wurtzite structure of ZnO, which consists of two interpenetrating hexagonal close packed sublattices (symmetry group P62mc (186)). According to Equation (1), the nanoparticles consisted of a single ZnO phase (100%). Using the Scherrer equation, the average size of the synthesized zinc oxide nanoparticles was calculated as 44.6 ± 5 nm. The change in the full width at half maximum (FWHM) value of the main diffraction lines on X-ray diffraction patterns depended directly on the degree of crystallinity (DC) of the samples. The width of the registered FWHM lines was calculated by approximating the lines on the diffractogram with the necessary number of symmetric pseudo-Voigt functions, which, in the long run, allowed us to characterize the perfection of the crystalline structure and to evaluate the DC [36,40]. The degree of crystallinity of biogenic ZnO was calculated to be 91.2%.

**Figure 3:**
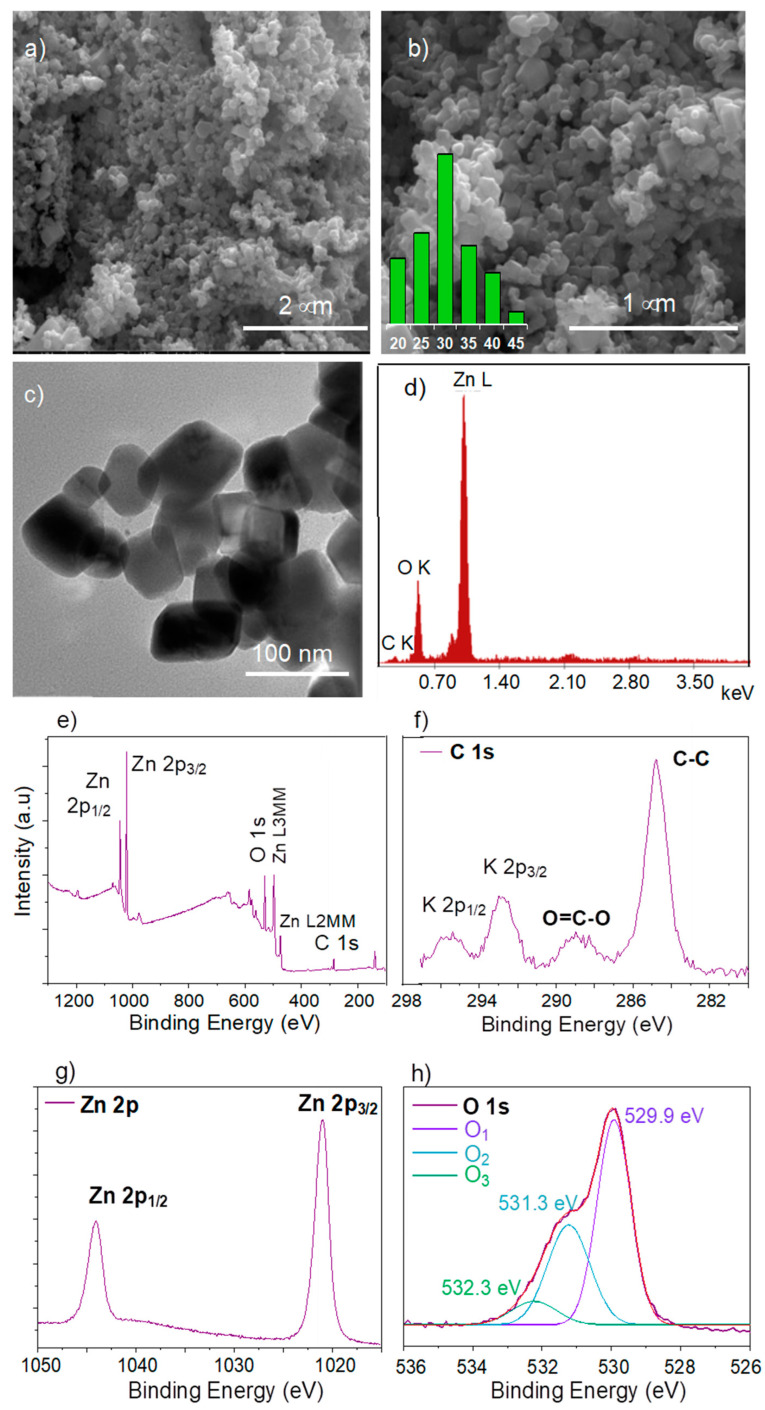
Electron microphotographs at 40,000× (**a**); and 80,000× magnification (the inset size distribution histogram was calculated from SEM analysis at 100,000× magnification) (**b**). TEM image (**c**) and Energy dispersion spectrum (EDS) of ZnO nanopowders (**d**). X-ray photoelectron spectroscopy of ZnO nanoparticles: survey wide spectrum (**e**) and C 1s (**f**), high-resolution spectra of Zn 2p (**g**) and O 1s (**h**).

The surface morphology of the nanoparticles was examined by scanning electron microscopy (Figure 3a,b). The microphotographs show ZnO nanoparticles of spherical and cubic geometry, with voids and pores between them, the formation of which can be explained by the large amount of hot gases released from the reaction mixture during synthesis via wet combustion [41]. Nanoparticle crystallites were interconnected with each other through these pores of different sizes and shapes. The average nanoparticle size was determined to be around 31 ± 5.5 nm. The majority of the ZnO NPs were ~30 nm in size and presented a narrow size distribution in the range of ~20–45 nm, as seen from the size distribution histogram inserted in Figure 3b. TEM analysis presented in Figure 3c confirmed the observations obtained by SEM. As can be seen in Figure 3c, spherical and cubic ZnO nanoparticles of approximately 30 nm in size were observed in the TEM analysis.

**Figure 4:**
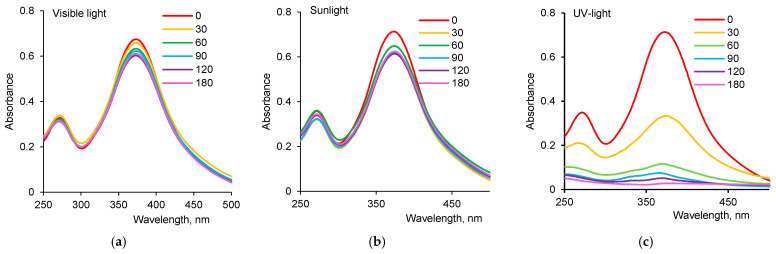

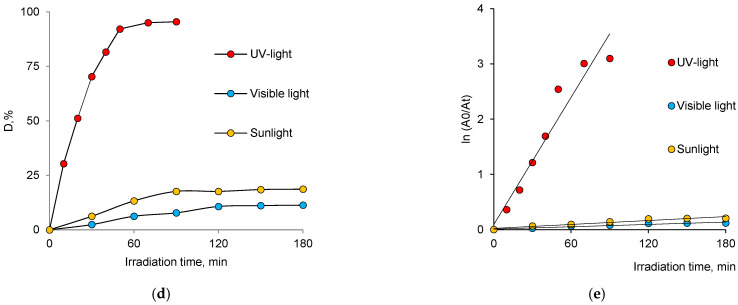
Absorption spectra of AY (1.0 mg/L) as a function of time in the presence of ZnO oxide nanoparticles (dye concentration—10.0 mg/L, loaded catalyst—50 mg) using visible light (**a**), sunlight (**b**) and UV-light (**c**), dye degradation efficiency (D, %) under different light sources (**d**) and Langmuir–Hinshelwood plot for the degradation of AY under different light sources (**e**).

As can be seen in Figure 3d, by examining the chemical composition through energy distribution analysis, in addition to Zn (weight: 79.2%, atomic: 46.9%) and O (weight: 17.4%, atomic: 43.0%) elements, some C atoms were detected in the structure of nanoparticles, which were attributed to the *Seratula coronata* L. extract used as a stabilizing and reducing agent during the synthesis, in agreement with previous works [42,43]. ZnO nanoparticles were also analyzed by the XPS method. The main advantage of XPS, compared to other techniques, is that it allows the elucidation of a chemical structure, as it provides information about precise determination of the oxidation state and chemical environment [36]. Therefore, this technique could be used to confirm the purity, but more importantly, the chemical composition and oxidation states of the ZnO nanoparticles we synthesized using plant extracts [44]. The wide energy range X-ray scan of biogenic ZnO, in Figure 3e, shows oxygen and the corresponding metal atom (Zn) present in its chemical composition. In addition, carbon (8.2%) was detected in the survey scan of ZnO. The high-resolution C 1s spectra in Figure 4f indicated a trace amount of K (around 1.0%) as impurities. The C 1s spectrum presented a main peak at 284.5 eV attributed to carbon containing C-C/C-H bonds, and another component at higher energy, of approximately 288.1 eV, assigned to carbon atoms containing O–C=O binding. Both peaks of the C 1s spectrum were attributed to the organic residues of plant extracts, in agreement with the EDS result. Figure 3g shows the high resolution XPS spectrum of the Zn 2p region. From this figure, the Zn 2p core-level of ZnO NPs had two fitting peaks located at approximately 1044.6 and 1021.5 eV, which were attributed to Zn 2p_1/2_ and Zn 2p_3/2_, respectively. Furthermore, the XPS spectrum of the O 1s region of ZnO nanoparticles (Figure 3h) showed an asymmetrical peak that could be deconvoluted into three near-Gaussian sub-peaks at 529.9, 531.3, and 532.3 eV. The peak at 529.9 eV (O_1_) was attributed to fully oxidized O^2-^ ions in a wurtzite ZnO lattice, while the peak at 531.3 eV (O_2_) was assigned to oxygen vacancies. The peak at the highest binding energy (O_3_, 532.4 eV) was associated with the presence of loosely bound oxygen on the surface, such as hydroxyl groups of H_2_O integrated into the material. The results from the core-level Zn 2p and O 1s spectra were clearly in very good agreement with the previous data and confirmed the ZnO structure [45,46,47,48]. In the light of the results discussed above, it could be concluded that the synthesis of biogenic ZnO NPs was accomplished successfully and the desired structures were obtained.

### 3.2. Study of the Photocatalytic Degradation Reaction of AY in the Presence of ZnO Nanoparticles

Figure 4a–c shows the spectroscopic monitoring of the optical density of AY degradation reaction under different light sources. Using the maximum absorbance at 373 nm in Formula (2), the calculated variation in dye degradation efficiency, depending on reaction time (Figure 4d), demonstrated that the highest efficiency of biogenic ZnO nanoparticles was observed when using UV-light. At 60 min after the beginning of the reaction, more than 95% of the initial dye was degraded under UV-light, while only 13.2 and 6.2% of AY were removed from the reaction mixture when sunlight and visible light were used for the same period of time, respectively.

The values of the reaction rate constants calculated from the kinetic curves in Figure 4e were 0.0019 min^−1^, 0.0017 min^−1^ and 0.0204 min^−1^, respectively, when conducting the reaction under sunlight, visible light and UV-light, indicating that the degradation reaction was significantly faster under UV light.

**Figure 5:**
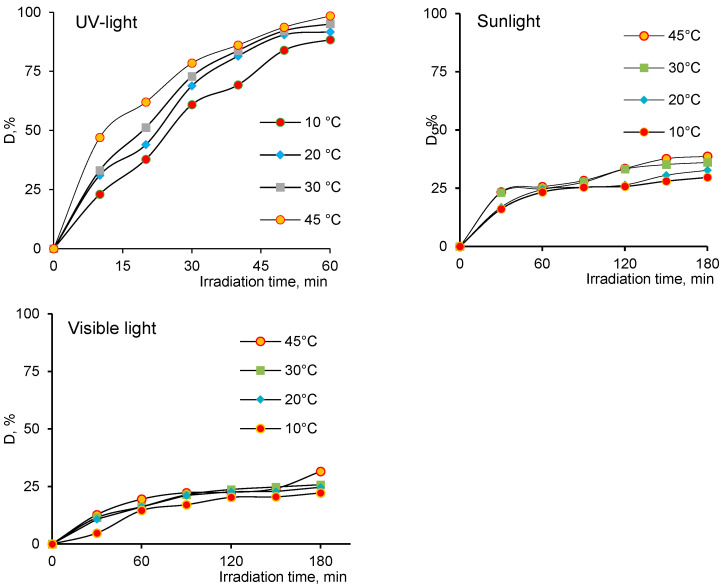
Influence of temperature on the degree of AY degradation (D, %) depending on the radiation source (nanoparticle mass—50 mg, AY concentration—10.0 mg/L).

Another important parameter affecting the reaction kinetics was temperature, which is typically described via the Arrhenius law [49]. The effect of temperature on the degradation efficiency of AY was studied in the temperature regime of 10–45 °C. As can be seen from the graphical dependencies of the dye degradation efficiency (D, %) at different temperature regimes in Figure 5, biogenic ZnO nanoparticles effectively accelerated the degradation of AY, even at 10 °C under UV light. After 60 min of reaction, about 88% of the dye was degraded at 10 °C, and D showed an overall increasing trend with temperature. Whereas, when AY was exposed to sunlight, even at elevated temperatures, the D did not exceed 40%, and the maximum D value at 45 °C was only 31% when visible light was used.

The activation energy (*E_a_*) was calculated using the Arrhenius Equation (5):

(5) $$\ln\;k=\ln A-\frac{E_{a}}{RT},$$

where *k* is the rate constant, min^−1^; *A* is the pre-exponential multiplier; *E_A_* is the activation energy, J/mol; *R* is the gas constant, equal to 8.314 J/mol·K; *T* is the temperature, K.

**Figure 6:**
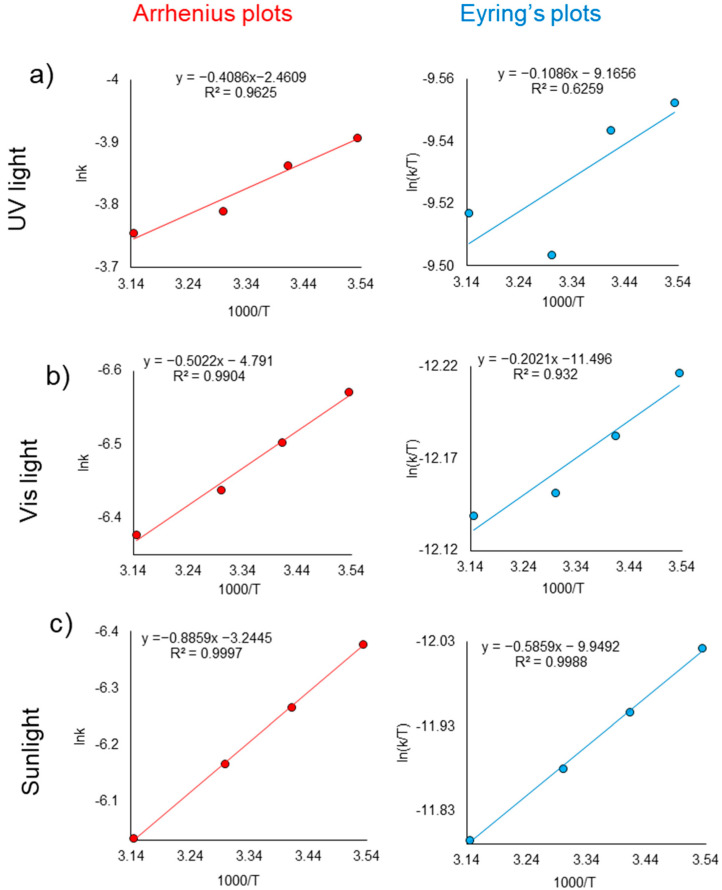
Arrhenius plots (**left**) and Eyring’s plots (**right**) for determining the thermodynamic characteristics of AY degradation reaction under (**a**) UV light, (**b**) visible light and (**c**) sunlight.

Δ*E_a_* was determined graphically from the dependence of ln *k* − (1000/*T*), in Figure 6. For all light sources used in the study, the obtained dependencies were characterized by a high coefficient of determination, *R*^2^. Eyring diagrams were plotted (Figure 6) [50] for the graphical determination of the activation enthalpy and entropy of the studied reaction. The calculated thermodynamic functions are summarized in Table 1. Clearly, the positive ΔH value referred to an endothermic reaction and the positive ΔG indicated that the photodegradation of AY was not spontaneous under any type of radiation. While ordinary catalyzed reactions were limited to negative ΔG values, i.e., spontaneous reactions, both cases were possible for photocatalytic reactions. In other words, a photocatalyst could drive reactions with both positive and negative ΔG [51,52].

As can be seen from the obtained data in Table 1, the degradation had the lowest activation energy Δ*E_a_* when AY was exposed to UV light. As discussed earlier, the degradation of AY was also kinetically faster under UV light; therefore, it was decided to use UV radiation in further studies, as the catalytic degradation of AY dye was the most effective compared to solar and visible light. Since photocatalytic reactions proceeded even when overall ΔG was positive, their rate might not be controlled by Δ*E_a_*. Actually, Δ*E_a_* values measured in a variety of photocatalytic reactions have been reported to be around 10 kJ/mol [53], much smaller than those of ordinary chemical reactions. In the field of photochemistry, the activation energy and rate determining step are rarely discussed because, generally speaking, photochemical reactions are not series reactions involving a rate determining chemical step, and, thus, the observed relatively low activation energy usually corresponds to a physical step, e.g., diffusion depending on the reaction temperature. Considering that photocatalysis is essentially a photochemical reaction, it makes no sense to discuss the chemical step that determines the rate. However, there are many publications that clearly show the temperature dependence of photo-catalytic reactions and use Arrhenius law to calculate the apparent activation energies, Δ*E_a_* [51,52,53]. Although these are modest values compared to typical catalytic processes, Δ*E_a_* should not be overlooked either.

**Figure 7:**
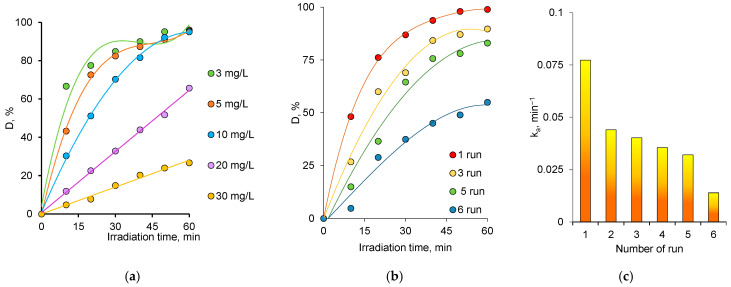
Change of degree of degradation, D, of AY depending on the dye concentration under UV-light (**a**). Variation of D (**b**) and reaction rate constant, ka (**c**) of AY in repeated uses of ZnO catalyst over 6 consecutive test cycles.

Examining the effect of dye concentration on the degree of decomposition, D, allowed us to determine the concentration range in which the catalyst could be further used effectively. The change in D value, depending on the concentration of AY, is shown in Figure 7. Biogenic ZnO nanoparticles provided almost 100% degradation of AY in the concentration range of 3–10 mg/L. When dye solutions at concentrations of 20.0 and 30.0 mg/L were exposed to UV-irradiation for 60 min, 65.7% and 26.7% degradation were obtained, respectively.

Stability and reusability are of great importance for the practical application of catalysts. In this study, in order to evaluate the stability of the properties of catalysts based on ZnO nanoparticles, 6 consecutive test cycles were carried out to decompose AY at a concentration of 10.0 mg/L (Figure 7b,c). After the 6th test cycle, the value of the D decreased from 98.97% to 55.0% (Figure 7b). In order to evaluate the catalyst activity, we calculated the rate constant, *k*, of the reaction in each test cycle. The value of the reaction rate constant decreased by 43% after the second test cycle and by more than 82% at the end of the 6th cycle. This finding suggested that decreasing degradation efficiency over the recycling experiments was mainly caused by the deactivation of catalytic sites. A comparison of existing catalysts in the literature developed for the photocatalytic degradation of AY with the performance the biogenic ZnO nanoparticles we synthesized in this study is presented in Table 2. When the data are carefully examined, the achievement of a very high degradation efficiency of 98% at comparable levels of the parameters affecting degradation, such as initial concentration of AY, contact time and amount of catalyst, indicated that the performance of the synthesized nanoparticles could compete with existing alternatives or be even higher.

### 3.3. Kinetic and Isotherm Study of AY Removal by Sorption

**Figure 8:**
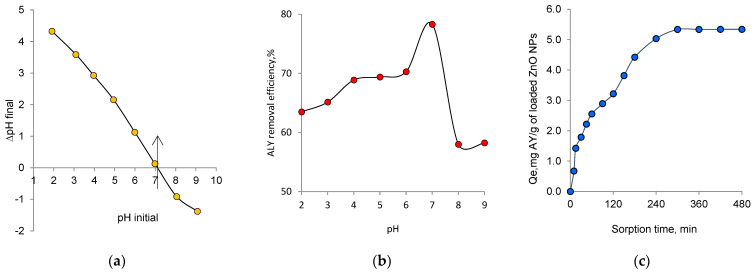

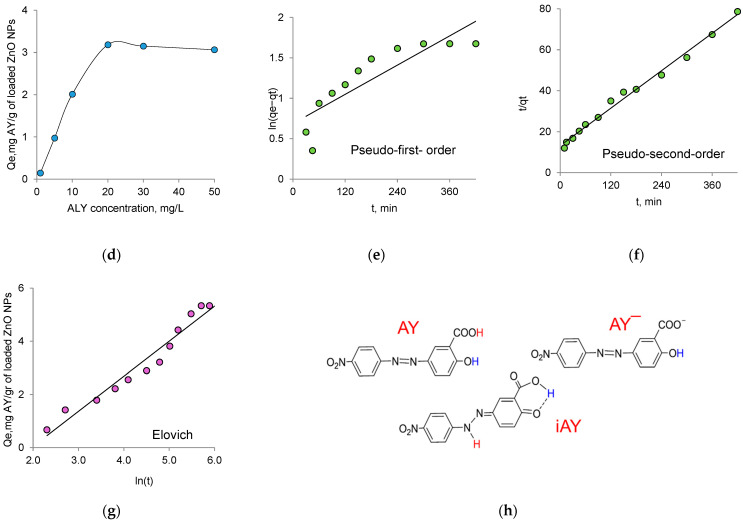
Point zero charge (pHPZC) plot (**a**), removal of AY as a function of solution pH (**b**) (AY concentration: 20 mg/L; biogenic ZnO NPs: 50 mg; contact time: 300 min); effect of contact time (**c**) and initial dye concentration (**d**) on the sorption of AY; kinetic model plots of pseudo-first-order (**e**), pseudo-second-order (**f**) and Elovich (**g**). (pH = 7.0, biogenic ZnO NPs: 50 mg; contact time: 120 min), (**h**) different forms of AY depending on solution pH.

It has been shown in detail in the previous section that ZnO nanoparticles catalyze the photodegradation of AY under an applied light source, especially UV light. Besides a degradation process, ZnO nanoparticles may also be effective in adsorption of AY, due to their high surface area. Therefore, in addition to their catalytic activities, in this section, we examined the performance of the biogenic nanoparticles we synthesized in the removal of AY by sorption. The point of zero charge pH (pH_PZC_) defines the pH of the solution at which the net surface charge of adsorbent is equal to zero. The study of pH_PZC_ is important to identify the adsorption mechanism and, thus, to explain the nature of the interactions [63]. Figure 8a shows the plot of pH_final_ versus pH_initial_. It can be seen from this figure that the pH_PZC_ value obtained for biogenic ZnO NPs was approximately 7.1, which was consistent with the value obtained by Katarina et al. [64]. Meanwhile, Leiva et al. [17] and Chauhan et al. [65] reported pH_pzc_ values of 6.21 and 7.5 respectively. The differences observed with existing studies might be a consequence of the synthesis method, the aggregation of NPs, and the presence of impurities, among other factors [17]. The effect of solution pH on dye adsorption is presented in Figure 8b. The neutral condition favored AY removal, and the maximum adsorption was observed at pH 7.0 (78.3% removal after 120 min), consistent with the previously reported optimum pH [59].

AY is an azo dye with an orthohydroxybenzoic group (Figure 8h). Its pKa value is 11.0 and its colour is yellow at pH 10.1 and turns to red at pH 12 [66,67]. The carboxyl group of AY is capable of deprotonation in the aqueous solution. Therefore, the anion (AY^−^) and neutral (AY) forms of alizarin yellow R are likely to coexist. On the other hand, the deprotonation of AY may also result in a hydrazone tautomer (iAY), via an attachment of the departed proton to the azo group [68]. At pH > 7.1, the adsorption efficiency decreased because the negatively charged ZnO NPs provided fewer effective sites for adsorption, due to increased repulsive forces. Thus, the removal of AY from aqueous solutions should not be conducted at a pH higher than 7.1. Generally, at pH < pH_pzc_, the sorbent surface has positively charged. At lower pH values, the interaction between AY and ZnO NPs is expected to be greater, due to the protonation of the carboxyl groups, compared to the higher pH values, at which they are negatively charged [22]. On the other hand, at even lower pH, ZnO NPs are highly soluble in aqueous solution [17]. In the presence of these opposite effects, the optimum pH was determined as 7.1. The effect of contact time on the adsorption of AY was studied in the range of 10 min to 420 min using 50 mg adsorbent at 25 °C and at pH 7, which was determined as optimum (Figure 8c). As can be seen from this figure, the maximum removal efficiency was achieved within around 300 min and there was no change in the equilibrium sorption capacity (*Q_e_*) after 360 min. Therefore, 360 min was considered to be an effective equilibrium time for adsorption. The *Q_e_* of biogenic ZnO NPs was found to be 5.34 mg AY/g.

In general, the initial concentration of the analyte is considered an important factor that can affect the adsorption process, creating the necessary driving force to overcome the resistance to mass transfer of AY between the aqueous solution and the sorbent surface [23]. According to the plot presented on the Figure 8d, the AY adsorption capacity increased from 0.15 to 3.2 mg/g when the initial concentration increased from 1 mg/L to 20 mg/L, and beyond this concentration it remained almost constant, due to the saturation of the active sites of the solid phase with dye molecules. Therefore, all further experiments were carried out with solutions at a feed concentration of 20 mg/L.

Adsorption kinetics studies are important to know the mechanism of adsorption and to design the treatment system. Three kinetic models, namely, pseudo-first-order, pseudo-second-order and Elovich models, were used to investigate the adsorption kinetics of AY on the biogenic ZnO NPs. Figure 8e–g shows the kinetic plots for the examined models. The correlation coefficients (*R*^2^), as well as the parameters calculated from the models, are summarized in Table 3. The pseudo-second-order kinetic model fitted the experimental data better (*R*^2^ = 0.99) than the pseudo-first-order-kinetic model. For the Elovich model, the initial sorption rate constant (*α*) of AY was higher than the desorption constant (*β*), confirming a high affinity of AY to the biogenic ZnO NPs [69]. The sorption capacity calculated by the pseudo-second-order model (6.56 mg/g) was in very good agreement with the experimental data. The applicability of the pseudo-second-order kinetic model for the biogenic sorbent led to the conclusion that chemisorption was the rate-determining step of the process, and the effect of the diffusion stage was insignificant.

**Figure 9:**
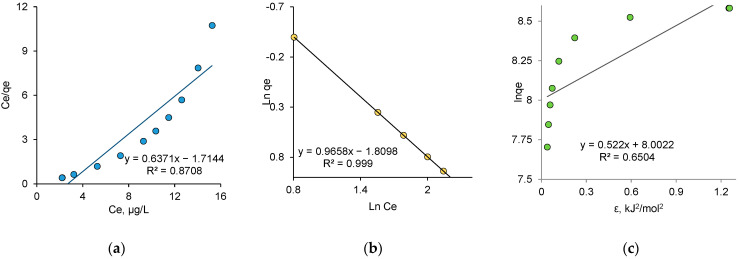
Fitted Langmuir (**a**), Freundlich (**b**) and DR (**c**) adsorption isotherms for AY adsorption on biogenic ZnO NPs.

Besides photocatalytic removal, one of the most popular and cost-effective techniques for the effective treatment of various water contaminants (dyes, heavy metals ions, pesticides, etc.) is adsorption. The correct understanding and interpretation of adsorption isotherms is a key step for the development of new types of effective adsorbents [70]. The equilibrium in the adsorption system depends on the nature of the interactions between adsorbent and adsorbate [71]. Well-known adsorption models, namely Langmuir, Freundlich and Dubinin–Radushkevich (DR), describe these interactions in different ways. The linearized equation forms of all studied isotherm models and the determined isotherm parameters are presented in Table 4. The Langmuir model describes the equilibrium between the adsorbate and adsorbent, where the adsorbate adsorption is limited to one molecular layer at, or before, a relative pressure of unity is reached [72]. Langmuir parameters b and Q_0_ were calculated from the intercept and slope of the linear plot presented in Figure 9a.

The Freundlich model suggests neither homogeneous energy sites nor limited adsorption levels. This means that the Freundlich model can describe experimental data of the adsorption isotherm whether adsorption occurs on homogeneous or heterogeneous sites, and is not controlled by the formation of the monolayer. According to this model, adsorption centers have different energy values. Therefore, the active sorption centers with maximum energy are filled first, then the process continues with the others. Figure 9b shows experimental data adapted to the linear Freundlich equation. The constant “n” is an empirical parameter related to the adsorption strength, which varies depending on the heterogeneity of the adsorbent. In our study, value of the adsorption intensity (n) was calculated as 1.04. According to Freundlich theory, n can also be used to determine whether adsorption is favorable. When n > 1, adsorption is favorable, n = 1 indicates a linear adsorption, and when n < 1, adsorption is unfavorable [73]. The Freundlich isotherm constant for adsorption capacity (*k*_*F*_) was calculated as 6.11 mg/g. The correlation coefficient (*R*^2^) was equal to 0.99, indicating very good agreement of the experimental data with the Freundlich adsorption isotherm. It was obvious that the Freundlich isotherm model described the adsorption process better than the Langmuir model. The applicability of the Freundlich model to the absorption of AY by biogenic ZnO nanoparticles indicated that the adsorption centers on the surface of nanoparticles were energetically nonequivalent and the surface of these natural sorbents was inhomogeneous.

Although the Langmuir and Freundlich models are widely used to explain the sorption process, they do not provide much information about the adsorption mechanism. Therefore, the equilibrium data were tested using the DR isotherm model to examine the mechanism of the adsorption process. The DR isotherm model (Figure 9c) is often used to identify physical or chemical adsorption, as the DR constant *β* is used to determine the sorption energy. Physical adsorption is believed to occur when the adsorption energy is less than 8 kJ/mol. At adsorption energies between 8 to 16 kJ/mol, it is assumed that chemisorption takes place. From the application of the DR model, an adsorption energy of 0.81 kJ/mol was obtained for the adsorption of AL dye on biogenic ZnO NPs, indicating a process through physical adsorption. However, a very low *R*^2^ value (0.65) indicated that the experimental data poorly fit the DR adsorption isotherm and, therefore, the obtained values might fall outside the confidence interval. In other studies, for example, using multi-walled carbon nanotubes (MWCNTs) encapsulated by polyaniline (PANI), the adsorption free energy was calculated as 14.14 kJ/mol, and the interactions between AY dye and the composite were of chemical type [74]. A comparison of the adsorption capacities of different AY adsorbents and biogenic ZnO attained in this study is presented in Table 5. It should be noted that it is rather difficult to directly compare the data of various studies, as some determining parameters, such as amount of loaded sorbent, agitation speed, pH and temperature, are not exactly the same. Overall, the adsorption capacity appeared to be low, but still, given the sustainability of the synthesis method and their ability to simultaneously photo-catalyze the degradation of AY, it could be concluded that biogenic ZnO nanoparticles might be advantageous and effective alternatives for AR removal.

## 4. Conclusions

In this study, biogenic ZnO nanoparticles were successfully synthesized using an eco-friendly wet combustion technique with *Serratula coronata* L. plant extract as the reducing and stabilizing agent, and, then, characterized by XRD, SEM, XPS, TEM and EDS methods, which confirmed the formation of monoclinic ZnO phase of high purity and narrow size distribution.

The as-prepared NPs were examined as to their effectiveness as photocatalyst/sorbent and their activities were evaluated in the removal of alizarin yellow R (AY) dye. The influences of light source, dye concentration, pH, temperature and reusability on the efficiency of the biogenic photocatalyst in the photodegradation of AY were studied. The results revealed that in the presence of synthesized ZnO NPs, the highest removal efficiency of AY was achieved when exposed to UV-irradiation (98.5%), whereas the removal efficacy efficiency remained at 31.6% and 38.7% under visible-light and sun-light, respectively.

The kinetics of the reaction was also evaluated, showing that the photocatalytic degradation of AY dye follows the Langmuir-Hinshelwood kinetics. The effect of temperature in the range of 10–45 °C was studied and activation energy was found to be 3.4 kJ/mol for UV-irradiation, while it was calculated as 4.18 and 7.37 kJ/mol under visible-light and sun-light, respectively.

Besides their photocatalytic activities, the performance of the synthesized ZnO nanoparticles was also investigated for the removal of AY dye from aqueous solutions by sorption. Examination of different pHs showed that the maximum removal efficiency was at 7.0. The adsorption process followed pseudo-second-order-kinetics with a correlation coefficient of 0.99. Adsorption data were best supported by the Freundlich model with maximum adsorption capacity of 6.11 mg/g, in accordance with the experimentally obtained values. The value of *E*_*DR*_ obtained using the Dubinin-Radushkevich isotherm model suggested that the adsorption process occurred by a physical mechanism rather than chemical adsorption. The results show that biogenic nanoparticles with both photocatalytic activity and sorption capacity can provide an efficient way to remove toxic dyes from water.

## Figures and Tables

**Table 1:** Thermodynamic parameters of the degradation of AY.

Type of Radiation	Δ*E_a_*, kJ/mol	ΔH, kJ/mol	ΔS, J/(mol·K)	∆G, kJ/mol (283 K)
UV light	3.40	0.90	−0.27	87.96
Visible light	4.18	1.68	−0.29	84.64
Sunlight	7.37	4.87	−0.28	76.97

**Table 2:** Comparison of some properties and catalytic activities of different types of catalysts in the AY degradation reaction.

Catalyst	Nano-catalyst Test Conditions					D_max_, %	ka, min^−1^	*E_a_*, kJ/mol	Ref.
	Light Source	ALY, mg/L	Contact Time, min	Catalyst Dosage, mg	*T*, °C				
NiFe_2_O_4_@AC/UiO-66(Zr)	Visible light	50.0	180.0	50.0	25.0	75.1	0.007	-	[54]
WO_3_ doped Zn	Nd:YAG Laser	100.0	60.0	400	-	100.0	0.197	-	[55]
Sn-CCMN composite	UV-light	5.0	120	200.0	-	91.5	0.039	-	[56]
Ce^3+^/TiO_2_	UV-light	5.0	120	50.0	25.0	38.6	0.004	-	[57]
Biogenic ZnO NPs (cow dung)	UV-light	5.0	100	15.0	-	75.6	0.014	-	[58]
	Visible light	5.0	100	15.0	-	19.0	-	-	
SnO_2_/CeO_2_ nano-composite	UV-light	25.0	90	150.0	-	91.1	-	-	[59]
ZnO	UV-light	40.0	120	300.0	40.0	92.5	-	-	[60]
Fe NPs	Sunlight	100.0	2520	100.0	25.0	92.51	0.065	10.99	[61]
TiO_2_-ZrO_2_	Sunlight	10.0	240	200	-	68.0	-	-	[62]
Biogenic ZnO NPs (*Serratula coronata* L.)	Visible light	10.0	180	50.0	45.0	31.6	0.002	4.18	This study
	Sunlight					38.7	0.002	7.37	
	UV-light					98.5	0.020	3.40	

**Table 3:** Parameters calculated from various kinetic models (initial AY concentration: 20 mg/L, adsorbent dose: 50 mg, pH: 7.0).

Kinetic Model	General Equation	Linearized Equation	Biogenic ZnO NPs	
			Model Parameters	Value
Pseudo-first-order	$\frac{dq_{t}}{d_{t}}=k_{1}\left(q_{e}-q_{t}\right)$	$\ln\left(q_{e}-q_{t}\right)=\ln\;q_{e}-k_{1}t$	$k_{1}$, min^−1^	0.003
			$q_{e}$, mg/g	1.0
			*R* ^2^	0.75
Pseudo-second-order	$\frac{dq_{t}}{t}=k_{2}{\left(q_{e}-q_{t}\right)}^{2}$	$\frac{t}{q_{t}}=\frac{1}{k_{2}q_{e}^{2}}+\frac{t}{q_{e}}$	$k_{2}$, g/mg·min	0.001
			$q_{e}$, mg/g	6.56
			*R* ^2^	0.99
Elovich	$\frac{dq_{t}}{t}=\alpha \;\exp\left(-\beta q_{t}\right)$	$q_{t}=\frac{1}{\beta }(\ln\alpha \beta )+\frac{1}{\beta }\ln\;t$	*α*, mg/g·min	0.84
			*β*, mg/min	0.76
			*R* ^2^	0.96

**Table 4:** Parameters calculated from various isotherm models (initial AY concentration: 20 mg/L, adsorbent dose: 50 mg, pH: 7.0).

Isotherm Model	Linearized Equation	Biogenic ZnO NPs	
		Model Parameters	Value
Langmuir	$\frac{C_{e}}{q_{e}}=\frac{C_{e}}{Q_{0}}+\frac{1}{Q_{0}b}$	$Q_{0}$, mg/g	1.57
		*b*, L/mg	0.91
		*R* ^2^	0.87
Freundlich	$\ln q_{e}=\ln k_{F}+\frac{1}{n}\ln C_{e}$	$k_{F}$, mg/g	6.11
		n	1.04
		*R* ^2^	0.99
Dubinin–Radushkevich	$\ln q_{e}=\ln Q_{d}-\beta \varepsilon^{2}$	*Q_d_*, mg/g	2.98
		β, mol^2^/kJ^2^	0.52
		$E_{DR}$, kJ/mol	0.98
		*R* ^2^	0.65

**Table 5:** Comparison of the AY adsorption capacity of *Serratula coronata* L. mediated ZnO NPs and some other available sorbents.

Adsorbent	Sorption Conditions			*Q_e_*, mg/g	Rate Constant $k_{2}$, g/mg·min	Ref.
	Initial Concentration of Adsorbate, ppm	Amount of Adsorbent Utilized, mg	Aliquot Volume, mL			
Fe_3_O_4_@PPY composite	50.0	49.5	20.0	34.1	0.012	[23]
MWCNTs/PANI composite	100.0	5.0	20.0	884.8	0.002	[74]
Mg_6_Al_2_OH_16_CO_3_	50.0	1000.0	25.0	26.3	0.071	[75]
CuFe_2_O_4_@graphene	50.0	500.0	30.0	98.0	1.534	[22]
CuFe_2_O_4_				145.0	0.063	
Biogenic ZnO	20.0	50.0	15.0	5.3	0.003	This study

## Data Availability

Not applicable.

## References

[1] Khan I., Saeed K., Khan I. (2019). Nanoparticles: Properties, applications and toxicities. Arab. J. Chem..

[2] Jeevanandam J., Barhoum A., Chan Y.S., Dufresne A., Danquah M.K. (2018). Review on nanoparticles and nanostructured materials: History, sources, toxicity and regulations. Beilstein J. Nanotechnol..

[3] Anu Mary Ealia S., Saravanakumar M.P. (2017). A review on the classification, characterisation, synthesis of nanoparticles and their application. IOP Conf. Ser. Mater. Sci. Eng..

[4] Sharma D., Kanchi S., Bisetty K. (2019). Biogenic synthesis of nanoparticles: A review. Arab. J. Chem..

[5] Patil S., Chandrasekaran R. (2020). Biogenic nanoparticles: A comprehensive perspective in synthesis, characterization, application and its challenges. J. Genet. Eng. Biotechnol..

[6] Gour A., Jain N.K. (2019). Advances in green synthesis of nanoparticles. Artif. Cells Nanomed. Biotechnol..

[7] Sharma R., Garg R., Kumari A. (2020). A review on biogenic synthesis, applications and toxicity aspects of zinc oxide nanoparticles. EXCLI J..

[8] Wiesmann N., Tremel W., Brieger J. (2020). Zinc oxide nanoparticles for therapeutic purposes in cancer medicine. J. Mater. Chem. B.

[9] Jiang J., Pi J., Cai J. (2018). The Advancing of Zinc Oxide Nanoparticles for Biomedical Applications. Bioinorg. Chem. Appl..

[10] Noman M.T., Amor N., Petru M., Mahmood A., Kejzlar P. (2021). Photocatalytic behaviour of zinc oxide nanostructures on surface activation of polymeric fibres. Polymers.

[11] Majumder S., Chatterjee S., Basnet P., Mukherjee J. (2020). ZnO based nanomaterials for photocatalytic degradation of aqueous pharmaceutical waste solutions—A contemporary review. Environ. Nanotechnol. Monit. Manag..

[12] Kadam V.V., Shanmugam S.D., Ettiyappan J.P., Balakrishnan R.M. (2021). Photocatalytic degradation of p-nitrophenol using biologically synthesized ZnO nanoparticles. Environ. Sci. Pollut. Res..

[13] Sugiyama M., Salehi Z., Tokumura M., Kawase Y. (2012). Photocatalytic degradation of p-nitrophenol by zinc oxide particles. Water Sci. Technol..

[14] Primo J.D.O., Bittencourt C., Acosta S., Sierra-Castillo A., Colomer J.-F., Jaerger S., Teixeira V.C., Anaissi F.J. (2020). Synthesis of Zinc Oxide Nanoparticles by Ecofriendly Routes: Adsorbent for Copper Removal From Wastewater. Front. Chem..

[15] Poguberović S.S., Krčmar D.M., Maletić S.P., Kónya Z., Pilipović D.D.T., Kerkez D.V., Rončević S.D. (2016). Removal of As(III) and Cr(VI) from aqueous solutions using “green” zero-valent iron nanoparticles produced by oak, mulberry and cherry leaf extracts. Ecol. Eng..

[16] Yuvaraja G., Prasad C., Vijaya Y., Subbaiah M.V. (2018). Application of ZnO nanorods as an adsorbent material for the removal of As(III) from aqueous solution: Kinetics, isotherms and thermodynamic studies. Int. J. Ind. Chem..

[17] Leiva E., Tapia C., Rodríguez C. (2021). Highly Efficient Removal of Cu(II) Ions from Acidic Aqueous Solution Using ZnO Nanoparticles as Nano-Adsorbents. Water.

[18] Pourmoslemi S., Mohammadi A., Kobarfard F., Assi N. (2016). Photocatalytic removal of two antibiotic compounds from aqueous solutions using ZnO nanoparticles. Desalin. Water Treat..

[19] Mirgane N.A., Shivankar V.S., Kotwal S.B., Wadhawa G.C., Sonawale M.C. (2021). Degradation of dyes using biologically synthesized zinc oxide nanoparticles. Mater. Today Proc..

[20] Chauhan A., Verma R., Kumari S., Sharma A., Shandilya P., Li X., Batoo K.M., Imran A., Kulshrestha S., Kumar R. (2020). Photocatalytic dye degradation and antimicrobial activities of Pure and Ag-doped ZnO using Cannabis sativa leaf extract. Sci. Rep..

[21] Khan M., Ware P., Shimpi N. (2021). Synthesis of ZnO nanoparticles using peels of Passiflora foetida and study of its activity as an efficient catalyst for the degradation of hazardous organic dye. SN Appl. Sci..

[22] Hashemian S., Rahimi M., Kerdegari A.A. (2016). CuFe2O4@graphene nanocomposite as a sorbent for removal of alizarine yellow azo dye from aqueous solutions. Desalin. Water Treat..

[23] Gholivand M.B., Yamini Y., Dayeni M., Seidi S., Tahmasebi E. (2015). Adsorptive removal of alizarin red-S and alizarin yellow GG from aqueous solutions using polypyrrole-coated magnetic nanoparticles. J. Environ. Chem. Eng..

[24] Al-Rubayee W.T., Abdul-Rasheed O.F., Ali N.M. (2016). Preparation of a Modified Nanoalumina Sorbent for the Removal of Alizarin Yellow R and Methylene Blue Dyes from Aqueous Solutions. J. Chem..

[25] Konyaeva E.A., Alentyeva O.G., Mizina P.G. (2019). Morphological and anatomical study of crown sawwort (*Serratula coronata* L.) herb. Pharmacy.

[26] Myagchilov A.V., Sokolova L.I., Gorovoi P.G., Dmitrenok P.S. (2017). New Flavonoids from Serratula coronata L.. Pharm. Chem. J..

[27] Myagchilov A.V., Sokolova L.I., Gorovoy P.G., Kechaikin A.A. (2020). Features of the composition of flavonoids in the crowned saw-wort (*Serratula Coronata* L.S.L.) Siberia and the Far East of Russia. Chem. Plant Raw Mater..

[28] Kasterova E., Zibareva L., Revushkin A. (2019). Secondary metabolites of some Siberian species of plants tribe Cynareae (Asteraceae). S. Afr. J. Bot..

[29] Ivaschenko I.V., Rakhmetov D., Vergun O.M. (2019). Biochemical features of the introduced population of Serratula coronata L. (Asteraceae) in Central Polissia of Ukraine. Plant Var. Stud. Prot..

[30] Mashentseva A.A., Aimanova N.A., Temirgaziev B.S., Zhumazhanova A.T., Tuleuov B.I. (2020). Photocatalytic Activity of Copper(II) Oxide Nanoparticles Synthesized Using Serratula Coronata L. Extract. Pet. Chem..

[31] Melentiyeva A.N., Chuchalin V.S., Burkova V.N. (2011). Technological methods of herbs Salsola collina Pall. efficient processing. Bull. Sib. Med..

[32] Muniz F.T.L., Miranda M.A.R., Morilla dos Santos C., Sasaki J.M. (2016). The Scherrer equation and the dynamical theory of X-ray diffraction. Acta Crystallogr. Sect. A Found. Adv..

[33] Zhao P., Lu L., Liu X., De la Torre A., Cheng X. (2018). Error Analysis and Correction for Quantitative Phase Analysis Based on Rietveld-Internal Standard Method: Whether the Minor Phases Can Be Ignored?. Crystals.

[34] Zdorovets M.V., Kozlovskiy A.L. (2019). Investigation of phase transformations and corrosion resistance in Co/CoCo2O4 nanowires and their potential use as a basis for lithium-ion batteries. Sci. Rep..

[35] Agrawal S., Singh N.B. (2016). Removal of arsenic from aqueous solution by an adsorbent nickel ferrite-polyaniline nanocomposite. Indian J. Chem. Technol..

[36] Mashentseva A.A., Barsbay M., Zdorovets M.V., Zheltov D.A., Güven O. (2020). Cu/CuO Composite Track-Etched Membranes for Catalytic Decomposition of Nitrophenols and Removal of As(III). Nanomaterials.

[37] Chikkanna M.M., Neelagund S.E., Rajashekarappa K.K. (2019). Green synthesis of Zinc oxide nanoparticles (ZnO NPs) and their biological activity. SN Appl. Sci..

[38] Talam S., Karumuri S.R., Gunnam N. (2012). Synthesis, Characterization, and Spectroscopic Properties of ZnO Nanoparticles. ISRN Nanotechnol..

[39] Okpashi I.W., Obi Bonaventure V.E., Okoro U.C. (2015). Synthesis and Characterization of Zinc Oxide (ZnO) Nanowire. J. Nanomed. Nanotechnol..

[40] Ida T., Ando M., Toraya H. (2000). Extended pseudo-Voigt function for approximating the Voigt profile. J. Appl. Crystallogr..

[41] Prashanth G.K., Prashanth P.A., Nagabhushana B.M., Ananda S., Krishnaiah G.M., Nagendra H.G., Sathyananda H.M., Rajendra Singh C., Yogisha S., Anand S. (2018). Comparison of anticancer activity of biocompatible ZnO nanoparticles prepared by solution combustion synthesis using aqueous leaf extracts of Abutilon indicum, Melia azedarach and Indigofera tinctoria as biofuels. Artif. Cells Nanomed. Biotechnol..

[42] Osuntokun J., Onwudiwe D.C., Ebenso E.E. (2019). Green synthesis of ZnO nanoparticles using aqueous Brassica oleracea L. var. italica and the photocatalytic activity. Green Chem. Lett. Rev..

[43] Kahsay M.H., Tadesse A., RamaDevi D., Belachew N., Basavaiah K. (2019). Green synthesis of zinc oxide nanostructures and investigation of their photocatalytic and bactericidal applications. RSC Adv..

[44] Venezia A.M. (2003). X-ray photoelectron spectroscopy (XPS) for catalysts characterization. Catal. Today.

[45] Al-Gaashani R., Radiman S., Daud A.R., Tabet N., Al-Douri Y. (2013). XPS and optical studies of different morphologies of ZnO nanostructures prepared by microwave methods. Ceram. Int..

[46] Barreca D., Gasparotto A., Maccato C., Maragno C., Tondello E. (2007). ZnO Nanoplatelets Obtained by Chemical Vapor Deposition, Studied by XPS. Surf. Sci. Spectra.

[47] Gandla S., Gollu S.R., Sharma R., Sarangi V., Gupta D. (2015). Dual role of boron in improving electrical performance and device stability of low temperature solution processed ZnO thin film transistors. Appl. Phys. Lett..

[48] Sahai A., Goswami N. (2015). Structural and optical investigations of oxygen defects in zinc oxide nanoparticles. AIP Conf. Proc..

[49] Bloh J.Z. (2019). A Holistic Approach to Model the Kinetics of Photocatalytic Reactions. Front. Chem..

[50] Hu Q., Liu B., Zhang Z., Song M., Zhao X. (2010). Temperature effect on the photocatalytic degradation of methyl orange under UV-vis light irradiation. J. Wuhan Univ. Technol. Sci. Ed..

[51] Ohtani B. (2010). Photocatalysis A to Z-What we know and what we do not know in a scientific sense. J. Photochem. Photobiol. C Photochem. Rev..

[52] Liu B., Wu H., Parkin I.P. (2020). New Insights into the Fundamental Principle of Semiconductor Photocatalysis. ACS Omega.

[53] Ohtani B. (2014). Revisiting the fundamental physical chemistry in heterogeneous photocatalysis: Its thermodynamics and kinetics. Phys. Chem. Chem. Phys..

[54] Elshamy O.A., El-Fawal E.M. (2021). Synthesis of NiFe2O4@AC/UiO-66(Zr) for Enhancement of the Photocatalytic Performance of Alizarin Yellow R Under Visible-light. ChemistrySelect.

[55] Seddigi Z.S. (2010). Removal of Alizarin Yellow dye from water using zinc doped WO 3 catalyst. Bull. Environ. Contam. Toxicol..

[56] Khan H., Khalil A.K., Khan A. (2019). Photocatalytic degradation of alizarin yellow in aqueous medium and real samples using chitosan conjugated tin magnetic nanocomposites. J. Mater. Sci. Mater. Electron..

[57] Lalliansanga, Tiwari D., Lee S.M., Kim D.J. (2021). New insights in photocatalytic removal of Alizarin Yellow using reduced Ce3+/TiO2 catalyst. Environ. Sci. Pollut. Res..

[58] Shubha J.P., Kavalli K., Adil S.F., Assal M.E., Hatshan M.R., Dubasi N. (2022). Facile green synthesis of semiconductive ZnO nanoparticles for photocatalytic degradation of dyes from the textile industry: A kinetic approach. J. King Saud Univ.-Sci..

[59] Hassan S.S.M., Kamel A.H., Hassan A.A., Amr A.E.-G.E., El-Naby H.A., Elsayed E.A. (2020). A SnO2/CeO2 Nano-Composite Catalyst for Alizarin Dye Removal from Aqueous Solutions. Nanomaterials.

[60] Abass A.K., Raoof S.D. (2016). Photocatalytic removal of alizarin yellow r from water using modified zinc oxide catalyst. Asian J. Chem..

[61] Ahmed A., Usman M., Yu B., Ding X., Peng Q., Shen Y., Cong H. (2020). Efficient photocatalytic degradation of toxic Alizarin yellow R dye from industrial wastewater using biosynthesized Fe nanoparticle and study of factors affecting the degradation rate. J. Photochem. Photobiol. B Biol..

[62] Ramamoorthy S., Das S., Balan R., Lekshmi I.C. (2021). TiO2-ZrO2nanocomposite with tetragonal zirconia phase and photocatalytic degradation of Alizarin Yellow GG azo dye under natural sunlight. Mater. Today Proc..

[63] Villabona-Ortíz Á., Figueroa-Lopez K.J., Ortega-Toro R. (2022). Kinetics and Adsorption Equilibrium in the Removal of Azo-Anionic Dyes by Modified Cellulose. Sustainability.

[64] Kataria N., Garg V.K. (2017). Removal of Congo red and Brilliant green dyes from aqueous solution using flower shaped ZnO nanoparticles. J. Environ. Chem. Eng..

[65] Chauhan A.K., Kataria N., Garg V.K. (2020). Green fabrication of ZnO nanoparticles using Eucalyptus spp. leaves extract and their application in wastewater remediation. Chemosphere.

[66] Pirillo S., Ferreira M.L., Rueda E.H. (2009). The effect of pH in the adsorption of Alizarin and Eriochrome Blue Black R onto iron oxides. J. Hazard. Mater..

[67] Masoud M.S., Elsamra R.M.I., Hemdan S.S. (2017). Solvent, substituents and pH effects towards the spectral shifts of some highly coloured indicators. J. Serbian Chem. Soc..

[68] Jiao X., Yu H., Kong Q., Luo Y., Chen Q., Qu J. (2014). Theoretical mechanistic studies on the degradation of alizarin yellow R initiated by hydroxyl radical. J. Phys. Org. Chem..

[69] Maslova M.V., Ivanenko V.I., Yanicheva N.Y., Mudruk N.V. (2020). Comparison of the sorption kinetics of lead(II) and zinc(II) on titanium phosphate ion-exchanger. Int. J. Mol. Sci..

[70] Ayawei N., Ebelegi A.N., Wankasi D. (2017). Modelling and Interpretation of Adsorption Isotherms. J. Chem..

[71] Russakova A.V., Altynbaeva L.S., Barsbay M., Zheltov D.A., Zdorovets M.V., Mashentseva A.A. (2021). Kinetic and isotherm study of as(Iii) removal from aqueous solution by pet track-etched membranes loaded with copper microtubes. Membranes.

[72] Liu L., Luo X.-B., Ding L., Luo S.-L. (2019). Application of Nanotechnology in the Removal of Heavy Metal From Water. Nanomaterials for the Removal of Pollutants and Resource Reutilization.

[73] Chen C., Zhao P., Li Z., Tong Z. (2016). Adsorption behavior of chromium(VI) on activated carbon from eucalyptus sawdust prepared by microwave-assisted activation with ZnCl 2. Desalin. Water Treat..

[74] Wu K., Yu J., Jiang X. (2018). Multi-walled carbon nanotubes modified by polyaniline for the removal of alizarin yellow R from aqueous solutions. Adsorpt. Sci. Technol..

[75] Al-Salihi K.J., Alfatlawi W.R. (2021). Synthesis and Characterization of Low-Cost Adsorbent and used for Alizarin Yellow GG and Alizarin Red S Dyes Removal from Aqueous Solutions. IOP Conf. Ser. Mater. Sci. Eng..

